# Resident and Nursing Home Factors Associated With Adherence to a Personalized Music Intervention: Secondary Analyses From Music & MEmory: A Pragmatic TRial for Nursing Home Residents With ALzheimer's Disease (METRIcAL)

**DOI:** 10.1155/jare/2679462

**Published:** 2025-05-02

**Authors:** Ryan A. Conard, Rosa R. Baier, Anthony Sisti, Laura Dionne, Ellen M. McCreedy

**Affiliations:** ^1^Brown University, Providence, Rhode Island, USA; ^2^Center for Long-Term Care Quality & Innovation, Brown University School of Public Health, Providence, Rhode Island, USA; ^3^Department of Health Services, Policy & Practice, Brown University School of Public Health, Providence, Rhode Island, USA; ^4^Department of Biostatistics, Brown University School of Public Health, Providence, Rhode Island, USA; ^5^Center for Gerontology & Healthcare Research, Brown University School of Public Health, Providence, Rhode Island, USA

**Keywords:** adherence, Alzheimer's disease and related dementias, embedded pragmatic clinical trial, music, nonpharmaceutical intervention, nursing homes

## Abstract

**Objectives:** Music offers a promising nonpharmacological alternative for managing agitation in people with Alzheimer's disease and other dementias (ADRD). We report resident and nursing home (NH) characteristics associated with uptake of a personalized music intervention.

**Design:** *Post hoc* analysis of a cluster-randomized embedded pragmatic clinical trial (ePCT) involving delivering resident-preferred music to manage agitated behaviors.

**Setting and Participants:** A total of 463 residents with ADRD in 27 NHs randomized to receive the intervention.

**Methods:** We obtained resident and NH characteristics from Minimum Data Set and Long-Term Care FocUS data. In addition, we created a study-specific engagement measure, which describes the proportion of enrolled residents in a given NH with any nursing staff use of the intervention. We used hierarchical models to estimate associations between resident and NH characteristics and (1) any exposure to the personalized music intervention and (2) minutes of music received per study day.

**Results:** This *post hoc* analysis included 463 residents from 27 NHs (mean age: 80 years (standard deviation, SD: 12.2), 68.5% female, and 25.3% Black or African American). Resident characteristics associated with a greater likelihood of any exposure to the music included being Black or African American (*p*=0.02). NH characteristics were associated with greater likelihood of any exposure included higher quality star ratings (*p*=0.01) and nursing staff engagement with the intervention (*p*=0.01). Among those exposed to the music, younger residents (*p*=0.02), Black residents (*p*=0.03), and those with less health instability (*p*=0.03) received greater doses. Residents living in NHs with high nursing staff engagement also received higher doses (*p* ≤ 0.001).

**Conclusions and Implications:** Black race was associated with a greater probability of exposure and more use of a personalized music intervention, after controlling for NH quality. Nursing staff engagement with a personalized music intervention increased uptake. These findings are useful for future ePCTs of behavioral interventions in NHs.

**Trial Registration:** Clinicaltrials.gov Identifier: NCT03821844

**Summary**

• Frontline nursing staff engagement increased exposure to a personalized music intervention for managing agitation in nursing home residents with dementia.

## 1. Introduction

Emotional and behavioral disturbances, including aggression and agitation, are some of the most prevalent neuropsychiatric symptoms in people living with Alzheimer's disease or related dementias (ADRDs) [1, 2] and can reduce quality of life and increase caregiver burden, particularly in nursing homes (NHs) [3]. Although antipsychotic medications are commonly used off label to manage dementia-related behaviors, they have black boxed warnings due to adverse side effects, including higher risks of falls, stroke, and death [3, 4]. Nonpharmacological approaches, such as reminiscence therapies, may alleviate behavioral symptoms without these risks [3, 5–8].

Despite preliminary efficacy evidence for some nonpharmaceutical or behavioral dementia interventions, many trials have failed to demonstrate effectiveness, in part due to low uptake [9–11]. In our embedded pragmatic clinical trial (ePCT), Music & MEmory: A Pragmatic TRial for Nursing Home Residents with ALzheimer's Disease (METRIcAL), we found that a personalized music intervention did not reduce the frequency of staff-reported agitated behaviors over the course of a week [12]. Our findings are consistent with a smaller randomized, controlled trial of a similar intervention [9], but contrasts with evidence from larger observational studies [13, 14]. Observational studies often analyze data from compilers or volunteers, creating potential selection biases [15]. In METRIcAL, 71% of targeted residents were exposed and, among those exposed, the median dose per day was 22.1 min (interquartile range: 7.0–65.2) [16]. Secondary analyses of pragmatic trial data may help us better understand selection to increase participant targeting and adherence in future trials.

In this secondary analysis of METRIcAL trial data, we examine resident and NH characteristics associated with (1) any exposure to the intervention, and (2) minutes of music received per day. We also include a study-specific, quantitative measure of nursing engagement, developed as part of a larger implementation evaluation [17–19].

## 2. Methods

### 2.1. Ethics

Due to the study being deemed minimal risk, the Brown University Institutional Review Board approved the study's conduct with a waiver of individual informed consent (protocol #1705001793).

### 2.2. Study Sample: Facilities and Participants

We conducted this *post hoc* analysis using data from METRIcAL, which was conducted in 54 NHs (27 treatment and 27 control) from four multistate corporations from June 2019 through February 2020. The full trial is described elsewhere [12, 20, 21], but, in brief, aimed to evaluate the effect of a personalized music intervention, Music & Memory, on agitated behaviors in NH residents living with ADRD. NH inclusion criteria included having at least 20 residents who were long-stay (i.e., living in the NH for 100 days, with no more than 10 days outside the facility), had a dementia diagnosis, and were not completely deaf (i.e., would be able to hear music). We also restricted NH eligibility based on geography, excluding sites on the West coast due to data collection barriers. Prior to randomization, corporate leaders excluded NHs with recent immediate jeopardy citations from the Centers for Medicare & Medicaid Services (CMSs) or other competing demands that would affect their ability to start the intervention. This prerandomization exclusion was at the discretion of corporate leaders; only a few NHs were excluded. After applying these exclusion criteria and allowing sites to opt-in, 54 NHs were enrolled and randomized to receive the intervention or a usual care control.

This analysis focuses on the 27 intervention NHs. Within each NH, staff chose resident participants who liked music and who exhibited dementia-related behaviors that could potentially be improved or managed with music. We followed all participants for 4 months. Per protocol, we excluded those who died or were discharged before the end of 4 months.

### 2.3. Intervention

The NH project champion, most often an activities staff member, identified the resident-preferred music through a trial-and-error process. The champion loaded the music on a personalized music device (i.e., an iPod). The champion was supposed to hand off the loaded iPod to the nursing staff to use when behaviors were likely, or at early signs of agitation. In practice, activities staff (the champions) were often the ones using the music with the residents. The music was provided via a personalized music device operated by the staff [20]. Researchers advised staff to provide participants with at least 30 min of music a day. Residents in the control group received standard care, which often included listening to ambient music or music as a group.

### 2.4. Outcome Variables

We calculated two outcome variables: (1) intervention exposure (yes/no) and (2) intervention dose, defined as the median number of minutes per exposure day spent listening to music during the intervention period. Among participants with reported music play time, we calculated dose by multiplying a song's duration by the number of times a song was played and averaging the total over the number of resident days enrolled in the study. We assigned participants with (1) missing start or end dates or (2) improbable high play times, defined as two standard deviations (SDs) above the mean (i.e., 17 h per day of music) a standard exposure time of 60 days (half of the potential follow-up window).

### 2.5. Resident Characteristics

Resident-level characteristics included demographics (age, sex, race (white, Black or African American, and Asian), and ethnicity (Hispanic, not Hispanic)), comorbid conditions (heart failure, hypertension, anxiety disorders, depression, bipolar disease, psychotic disorders, and asthma/chronic obstructive pulmonary disease (COPD)), physical functioning as assessed by the Minimum Data Set (MDS) Activities of Daily Living Scale (ADL; range 0–28, with 0 indicating no functional dependence and 28 indicating total functional dependence) [22], cognitive functioning as assessed by the MDS 3.0 Cognitive Function Scale (CFS; range 0–4, with higher scores indicating increased cognitive impairment) [23], mortality risk as assessed by the MDS 3.0 Changes in Health, End-Stage Disease and Symptoms and Signs Scale (CHESS; range 0–5, with higher scores indicate increased risk of mortality) [24], the frequency of agitated behaviors at baseline as assessed by the Agitated and Reactive Behavior Scale (ARBS; range 0–12, with higher scores indicating increased frequency of agitated and reactive behaviors) [25], and medication use in the past week (antipsychotic, antidepressant, antianxiety).

### 2.6. Facility Characteristics

NH-level characteristics included CMS 5 Star Quality and Staffing Ratings, ownership type (for profit and nonprofit), and total beds. Quality stars are based on performance across seven quality metrics describing the proportion of eligible residents with ADL decline, mobility decline, urinary tract infections and catheter use, pain, delirium, and pressure ulcers. Staffing stars are based on registered nursing hours per resident day and total direct care staffing hours per resident day. More information on the 5 Star Rating System is publicly available at https://cms.gov.

### 2.7. Facility Engagement Measure

We calculated a nursing engagement measure, defined as the proportion of enrolled residents in a given NH who had a nursing staff member report using the intervention at any point in the past 2 weeks (range 0–1.0, with tertiles representing low, medium, and high nursing engagement). This measure was obtained while researchers were interviewing direct care staff about resident behaviors in the past 2 weeks. Details about how the engagement measure was created and scaled have been published elsewhere [18].

### 2.8. Data Sources

We obtained resident-level characteristics from the MDS, routinely collected, standardized assessment of NH residents' cognitive and physical function, medication use, and relevant comorbidities. Facility nurses assess residents upon admission, quarterly, annually, with a change in condition, and at discharge. We obtained facility-level characteristics from the public use Long-Term Care FocUS (LTCFocUS) administrative data [26]. LTCFocUS data summarize assessments of staffing, ownership, and payment mix information for US NHs. Nursing engagement was assessed by asking staff if they had used the music with the resident in the past 2 weeks (self-report).

### 2.9. Statistical Analysis

We used hierarchical models to examine the association between resident- and facility-level characteristics and the two outcomes, any use of the music (exposure) and logged minutes of music received per exposed day (dose). Specifically, we relied on hierarchical logistic regression to estimate associations for any use of music, and a linear hierarchical model among those that had used the music to estimate these associations with the number of minutes per exposed day. We estimated the marginal effects of different levels of resident and facility characteristics on the probability of exposure to the intervention. We completed all modeling using STATA, Version 14 [28].

## 3. Results

**Figure 1:**
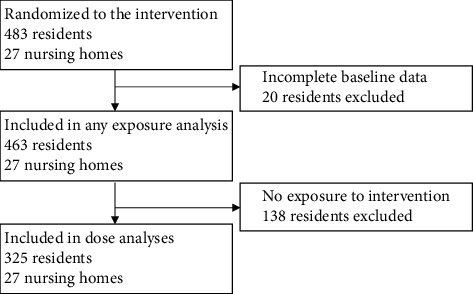
Consort diagram of nursing homes and residents randomized to receive the intervention and included in the *post hoc* analyses.

Across the 27 NHs, we enrolled 483 residents, 463 of whom had complete baseline data for the purposes of these analyses (Figure 1). On average, participants were 80.0 years of age (SD = 12.2), 72.1% White, and 68.5% female (Table 1). One-third of the participants (*n* = 154, 33.3%) were dependent on staff for most of their ADLs and two-thirds (*n* = 313, 67.6%) had moderate or severe cognitive impairment. One in four participants were taking antipsychotic medications (*n* = 118, 25.5%) and more than half were taking antidepressants (*n* = 264, 57.0%). The 27 treatment facilities had 100.7 beds (SD: 41.7), slightly above average quality ratings (3.5 out of 5 stars), and average staffing ratings (3.1 of 5 stars) (Table 2). There were more for-profit than nonprofit NHs randomized to the intervention (16 for-profit and 11 nonprofit NHs). On average, 45% of residents in treatment NHs had a nursing staff member engaged in delivering the music.

### 3.1. Any Exposure to Personalized Music Intervention

Overall, 70% of the targeted residents were exposed (325 out of 463 randomized to receive intervention). Resident-level characteristics associated with any exposure to the intervention included being Black or African American (*p*=0.02) (Table 3). NH characteristics associated with greater likelihood of any exposure included higher quality star ratings (*p*=0.01) and nursing staff engagement with the intervention (*p*=0.01). The intraclass correlation coefficient indicates that 7.7% of the residual variation was due to differences between NHs unexplained by available variables (95% CI: 0.03, 0.18).

### 3.2. Amount of Music Received, Among Exposed

Among those exposed, the mean minutes of music per exposed day was 61.1 min (SD: 108.0), and the median minutes of exposed day was 22.1 min (IQR: 7.0–65.2). Among those exposed to the music, younger residents (*p*=0.02), Black residents (*p*=0.03), and those with less health instability (*p*=0.03) received greater doses. Residents living in NHs with high nursing staff engagement also received higher doses of the music (*p* ≤ 0.001) (Table 3). The intraclass correlation coefficient indicated 5.4% of the residual variation in the second model was due to differences between NHs unexplained by available variables (95% CI: 0.01, 0.19).

### 3.3. Probability of Exposure Given Nursing Engagement and Race of Resident

**Figure 2:**
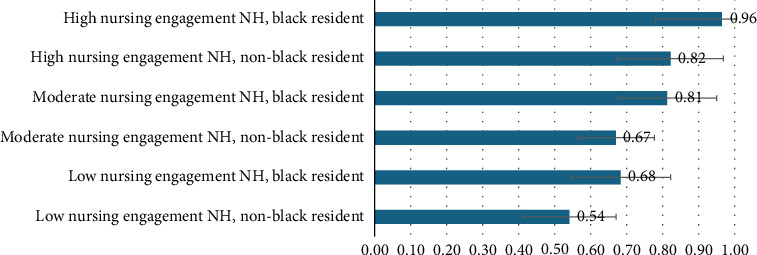
Adjusted probability of exposure to a music intervention, for Black and non-Black (White, Asian, and Hispanic) residents living in nursing homes with low, medium, or high nursing engagement. Abbreviation: NH, nursing home.

Figure 2 presents the probability of a resident being exposed to the music intervention based on the level of nursing engagement in their facility for Black and non-Black (White, Asian, and Hispanic) residents, while holding all other variables at their means. The adjusted probability of a non-Black resident in a low nursing engagement NH being exposed to the intervention was 0.54 (95% CI = 0.41–0.67), compared with 0.68 (95% CI = 0.54–0.82) for Black residents in similar NHs. The adjusted probability of a non-Black resident in a medium nursing engagement NH being exposed to the intervention was 0.67 (95% CI = 0.56–0.78), compared with 0.81 (95% CI = 0.68–0.95) for Black residents in similar NHs. The adjusted probability of a non-Black resident in a high nursing engagement NH being exposed to the intervention was 0.82 (95% CI = 0.67–0.97), compared with 0.96 (95% CI = 0.78–1.15) for Black residents in similar NHs.

## 4. Discussion

In this secondary analysis of data from an ePCT investigating a personalized music intervention for NH residents living with ADRD, we identified resident- and facility-level characteristics associated with greater probability of exposure to the intervention and receipt of a higher dose of music. Nursing home quality was a primary driver of whether or not a resident was exposed to the intervention, as was resident race. Race was also an important predictor of daily dose of music received. Importantly, after controlling for nursing home quality, engagement of frontline nursing staff with the intervention related to initial exposure and daily dose. Our results may inform others' implementation and evaluation of NH ePCTs.

The observation that exposure to our intervention depended, in part, on high nursing engagement is consistent with results from other NH ePCTs. In a 10-facility, single-state trial of the same personalized music intervention, Music & MEmory, Kwak et al. found that lack of buy-in from frontline nursing staff was the most frequently cited barrier to implementation [29]. Similarly, in a trial of a video-assisted advance care planning intervention, Loomer et al. found greater use of the intervention in NHs with higher rates of nurses attending check-in phone calls with researchers [10]. These findings highlight the importance of engaging nursing staff to achieve fidelity, independent of NH-level characteristics such as quality and staffing ratios. Nursing engagement in nonpharmaceutical dementia interventions may be especially critical to clinically target delivery to residents' unique needs (e.g., to use the music when dementia behaviors are likely or at early signs of agitation). However, this is challenging in NHs, where staff face competing priorities due to understaffing and the need to focus on regulatory compliance [30]. Researchers conducting ePCTs need creative solutions to engage staff, which should include direct compensation for time and promotional or career-enhancing training opportunities [31, 32].

The finding that Black residents were most likely to be exposed to the intervention than non-Black residents is contrary to prior implementation work and warrants further exploration. Previous studies suggest that Black residents living with ADRD are less likely to receive pragmatic interventions, as a result of disproportionately residing in lower-quality NHs [33]. Such facilities may not have the resources to implement novel quality improvement programs, because of lower levels of staffing or staffing instability and financial hardship [33, 34]. Thus, minoritized status and NH quality have often been predictors of low adherence in pragmatic trials. One possible explanation for our finding is that music may already be ingrained in the culture of these specific facilities with high proportions of minoritized residents, prior to the introduction of the intervention. Another benefit of the intervention is that it was personalized by staff at the NHs who often know, and share, a similar background to the residents. Personalization is time consuming, but may also circumvent more prescriptive protocols that are not developed for a community, by that same community.

As noted, our results reveal a pattern in the factors associated with music intervention exposure and dose, with exposure depending more on facility-level characteristics and dose more on resident-level characteristics. This highlights the complex interplay between facility- and resident-level factors that influence implementation. NH staff are in control of offering the intervention; NHs with certain cultures, policies, and staff training, as well as those with more resources, may have been more receptive to nonpharmacological approaches and ultimately more able or more likely to offer the intervention, leading to higher exposure rates [35, 36]. On the other hand, once offered, residents and their individual preferences, cognitive abilities, and clinical characteristics may influence how much they engage with the music once exposed, influencing dose. These explanations are speculative, and the true underlying mechanisms are unknown. Further investigation of this observed pattern may help in providing a general guide for factors that should be considered when implementing such interventions.

We note several limitations to consider when interpreting the results. First, given the small number of individuals from racial groups other than Black or White (*n* = 12), we had to combine residents of Asian and Hispanic descent with White residents and thus only explored race on the level of Black versus non-Black (mainly White) residents. However, Black or African American residents were well represented. Second, our data for the nursing engagement measure included staff self-report, which can introduce biases (e.g., recall and observation biases), especially since participants and NH staff were aware of the intervention. We aimed to minimize these biases through standardized measurements and training [37]. Third, our outcome variable exhibited a right-skewed non-normal distribution. We encountered several residents with exceptionally high minutes per day of listening time, often due to an impossibly low number of exposure days. We standardized their number of days exposed to 60 days, half of the follow-up window. In addition, we implemented two separate hierarchical models to address the high number of residents with no exposure and linearized the data in the second model to account for the non-normal distribution.

## 5. Conclusions and Implications

Our *post hoc* ePCT analysis shows higher-quality NHs were more likely to implement a personalized music intervention. However, after controlling for NH quality, Black residents were more likely to receive the intervention than non-Black residents, and nursing staff engagement with the intervention drove initial exposure and daily dose received by residents. These results highlight the value of capturing quantitative implementation measures to better understand variation between NHs and suggest potential factors to target to increase fidelity. Our finding that Black residents were more likely to be exposed to the intervention than non-Black residents warrants further exploration and research into culturally tailored protocols [27].

## Figures and Tables

**Table 1:** Resident characteristics at the baseline.

	Total, *N* = 463 residents	Exposed, *N* = 325 residents	Unexposed, *N* = 138 residents	*p* values
Demographics				
Age, mean (SD)	80.0 (12.2)	80.3 (11.3)	79.1 (14.0)	0.33
Females, *n* (%)	317 (68.5)	233 (68.6)	94 (68.1)	0.92
Race/ethnicity, *n* (%)				0.24
White, non-Hispanic	334 (72.1)	236 (72.6)	98 (71.0)	
Black, non-Hispanic	117 (25.3)	83 (25.5)	34 (24.6)	
Asian, non-Hispanic	4 (0.01)	1 (0.00)	3 (0.02)	
Hispanic	8 (0.02)	5 (0.02)	3 (0.02)	
Function				
Activities of daily living, *n* (%)				0.68
Severely impaired (≥ 21)	154 (33.3)	110 (33.9)	44 (31.9)	
Mild-moderately impaired (< 21)	309 (66.7)	215 (66.2)	94 (68.1)	
Cognitive function, *n* (%)				0.31
Cognitively intact	51 (11.0)	30 (9.2)	21 (15.2)	
Mildly impaired	99 (21.4)	70 (21.5)	29 (21.0)	
Moderately impaired	236 (51.0)	169 (52.0)	67 (48.6)	
Severely impaired	77 (16.6)	56 (17.2)	21 (15.2)	
CHESS score, *n* (%)				0.75
No health instability, 0	238 (51.4)	169 (52.0)	69 (50.0)	
Minimal/low instability, 1–2	153 (33.0)	107 (32.9)	46 (33.3)	
Moderate/high instability, 3	72 (15.6)	49 (15.1)	23 (16.7)	
Agitated and Reactive Behavior Scale, *n* (%)				0.39
0, no behaviors	356 (76.9)	248 (76.3)	108 (78.3)	
1-2, mild behaviors	71 (15.3)	54 (16.6)	17 (12.3)	
3+, moderate and severe behaviors	36 (7.8)	23 (7.1)	13 (9.4)	
Presence of comorbidities, *n* (%)				
Heart failure	67 (14.5)	50 (15.4)	17 (12.3)	0.39
Hypertension	365 (78.8)	257 (79.1)	108 (78.3)	0.84
Anxiety disorder	179 (38.7)	133 (40.9)	46 (33.3)	0.13
Depression	239 (51.6)	165 (50.8)	74 (53.6)	0.57
Bipolar disease	25 (5.4)	21 (6.5)	4 (2.9)	0.12
Psychotic disorder	54 (11.7)	42 (12.9)	12 (8.7)	0.20
Asthma/COPD	55 (11.9)	39 (12.0)	16 (11.6)	0.90
Medication use, *n* (%)				
Any antipsychotics in past week	118 (25.5)	86 (26.5)	32 (23.2)	0.46
Any antianxietals in past week	93 (20.1)	67 (20.6)	26 (18.8)	0.66
Any antidepressants in past week	264 (57.0)	183 (56.3)	81 (58.7)	0.64

*Note:* CHESS, changes in health, end-stage disease, and signs and symptoms.

Abbreviations: COPD, chronic obstructive pulmonary disease; SD, standard deviation.

**Table 2:** Nursing home characteristics at the baseline.

	Treatment nursing homes (*N* = 27)
CMS 5-star quality rating, mean (SD)	3.5 (1.3)
1-star, *n* (%)	2 (7.4)
2-star, *n* (%)	6 (22.2)
3-star, *n* (%)	5 (18.5)
4-star, *n* (%)	5 (18.5)
5-star, *n* (%)	9 (33.3)
CMS 5-star direct care staff rating, mean (SD)	3.1 (1.2)
1-star, *n* (%)	3 (11.1)
2-star, *n* (%)	5 (18.5)
3-star, *n* (%)	10 (37.0)
4-star, *n* (%)	5 (18.5)
5-star, *n* (%)	4 (14.8)
For-profit ownership, *n* (%)	16 (59.3)
Total beds, mean (SD)	100.7 (41.7)
Proportion of interviewed nursing staff engaged in the intervention, mean (SD)	0.45 (0.31)

*Note:* CMS, Centers for Medicare & Medicaid Services.

Abbreviation: SD, standard deviation.

**Table 3:** Resident and facility characteristics associated with any use of the personalized music intervention and dose received (among exposed).

	Any exposure to intervention (*N* = 463 residents), coefficient (95% CI)	Logged minutes per exposed day (*N* = 325 residents), coefficient (95% CI)
*Resident-level characteristics*		

*Demographics*		
Age	0.00 (0.00, 0.01)	−0.03 (−0.05, −0.00)^∗^
Female sex	−0.03 (−0.11, 0.06)	−0.06 (−0.51, 0.38)
Black race	0.14 (0.02, 0.26)^∗^	0.70 (0.09, 1.31)^∗^

*Function*		
Severely impaired activities of daily living score	0.06 (−0.04, 0.17)	0.09 (−0.42, 0.60)

*Cognitive function scale score (ref. no cognitive impairment)*		
Mild cognitive impairment	0.03 (−0.12, 0.18)	0.25 (−1.04, 0.54)
Moderate cognitive impairment	0.01 (−0.14, 0.15)	0.22 (−0.54, 0.99)
Severe cognitive impairment	0.10 (−0.09, 0.28)	−0.08 (−1.07, 0.91)

*CHESS score (ref. no health instability)*		
Minimal health instability	−0.06 (−0.17, 0.05)	0.64 (0.05, 1.23)^∗^
Low health instability	−0.10 (−0.27, 0.08)	0.34 (−0.58, 1.25)
Moderate/high health instability	−0.19 (−0.47, 0.09)	1.46 (−0.05, 2.98)

*Agitated and Reactive Behavior Scale (ref. no behaviors)*		
Mild behaviors	0.07 (−0.04, 0.19)	0.04 (−0.52, 0.61)
Moderate and severe behaviors	−0.03 (−0.19, 0.12)	0.30 (−0.50, 1.10)

*Presence of comorbidities*		
Heart failure	0.08 (−0.07, 0.23)	0.11 (−0.62, 0.86)
Hypertension	−0.02 (−0.12, 0.08)	0.18 (−0.32, 0.69)
Anxiety disorder	0.04 (−0.06, 0.14)	0.03 (−0.46, 0.52)
Depression	0.02 (−0.08, 0.13)	−0.01 (−0.53, 0.50)
Bipolar disease	0.16 (−0.02, 0.34)	0.31 (−0.54, 1.17)
Psychotic disorder	0.07 (−0.06, 0.21)	0.37 (−0.29, 1.03)
Asthma/COPD	−0.01 (−0.13, 0.12)	−0.26 (−0.88, 0.37)

*Medication use*		
Any antipsychotics in past week	0.01 (−0.09, 0.12)	0.36 (−0.17, 0.89)
Any antianxietals in past week	0.00 (−0.12, 0.11)	−0.18 (−0.76, 0.40)
Any antidepressants in past week	−0.01 (−0.11, 0.09)	−0.25 (−0.75, 0.26)

*Facility-level characteristics*		

*CMS 5-star quality rating (ref. 1-star)*		
2-star, *n* (%)	0.56 (0.19, 0.92)^∗^	0.27 (−1.36, 1.91)
3-star, *n* (%)	0.51 (0.13, 0.89)^∗^	0.38 (−1.33, 2.09)
4-star, *n* (%)	0.67 (0.27, 1.06)^∗^	0.53 (−1.23, 2.28)
5-star, *n* (%)	0.32 (0.02, 0.61)^∗^	0.28 (−1.04, 1.59)

*CMS 5-star direct care staffing rating (ref. 1-star)*		
2-star, *n* (%)	0.11 (−0.21, 0.43)	1.04 (−0.27, 2.35)
3-star, *n* (%)	−0.21 (−0.49, 0.06)	−0.07 (−1.22, 1.08)
4-star, *n* (%)	−0.27 (−0.57, 0.03)	0.62 (−0.67, 1.91)
5-star, *n* (%)	0.01 (−0.29, 0.30)	0.01 (−1.25, 1.23)
For-profit ownership	0.07 (−0.13, 0.27)	0.07 (−0.79, 0.92)
Total number of beds	0.00 (0.00, 0.00)	−0.00 (−0.00, 0.00)

*Nursing staff engagement with intervention (ref. low)*		
Moderate nursing staff engagement	0.13 (−0.04, 0.30)	0.59 (−0.15, 1.33)
High nursing staff engagement	0.28 (0.08, 0.48)^∗^	1.90 (1.06, 2.73)^∗^

Residual intraclass correlation (ICC)	0.08 (0.03, 0.18)^∗^	0.05 (0.01, 0.19)^∗^

*Note:* CHESS, changes in health, end-stage disease, and symptoms and signs scale; CMS, Centers for Medicare & Medicaid Services.

Abbreviation: CI, confidence interval.

^∗^ *p* < 0.05.

## Data Availability

Restrictions apply to the availability of these data. Analytic code files are available from the corresponding author upon request.
